# Development of an efficient Sanger sequencing-based assay for detecting SARS-CoV-2 spike mutations

**DOI:** 10.1371/journal.pone.0260850

**Published:** 2021-12-14

**Authors:** Ho Jae Lim, Min Young Park, Hye Soo Jung, Youngjin Kwon, Inhee Kim, Dong Kwan Kim, Nae Yu, Nackmoon Sung, Sun-Hwa Lee, Jung Eun Park, Yong-Jin Yang

**Affiliations:** 1 Department of Molecular Diagnostics, Seegene Medical Foundation, Seoul, Republic of Korea; 2 Department of Integrative Biological Sciences & BK21 FOUR Educational Research Group for Age-associated Disorder Control Technology, Chosun University, Gwangju, Republic of Korea; 3 Clinical Research Institute, Seegene Medical Foundation, Seoul, Republic of Korea; Defense Threat Reduction Agency, UNITED STATES

## Abstract

Novel strains of severe acute respiratory syndrome coronavirus 2 (SARS-CoV-2) harboring nucleotide changes (mutations) in the spike gene have emerged and are spreading rapidly. These mutations are associated with SARS-CoV-2 transmissibility, virulence, or resistance to some neutralizing antibodies. Thus, the accurate detection of spike mutants is crucial for controlling SARS-CoV-2 transmission and identifying neutralizing antibody-resistance caused by amino acid changes in the receptor-binding domain. Here, we developed five SARS-CoV-2 spike gene primer pairs (5-SSG primer assay; 69S, 144S, 417S, 484S, and 570S) and verified their ability to detect nine key spike mutations (ΔH69/V70, T95I, G142D, ΔY144, K417T/N, L452R, E484K/Q, N501Y, and H655Y) using a Sanger sequencing-based assay. The 5-SSG primer assay showed 100% specificity and a conservative limit of detection with a median tissue culture infective dose (TCID_50_) values of 1.4 × 10^2^ TCID_50_/mL. The accuracy of the 5-SSG primer assay was confirmed by next generation sequencing. The results of these two approaches showed 100% consistency. Taken together, the ability of the 5-SSG primer assay to accurately detect key SARS-CoV-2 spike mutants is reliable. Thus, it is a useful tool for detecting SARS-CoV-2 spike gene mutants in a clinical setting, thereby helping to improve the management of patients with COVID-19.

## Introduction

The World Health Organization (WHO) declared the coronavirus disease (COVID-19), which is caused by severe acute respiratory syndrome coronavirus 2 (SARS-CoV-2), as a global pandemic on March 11, 2020 [1]. SARS-CoV-2 is a highly transmissible virus and has a long incubation time before the manifestation of symptoms, such as fever, cough, shortness of breath, and diarrhea [2]. SARS-CoV-2 has a single-stranded, positive-sense RNA genome of approximately 29.9 kb, which encodes several proteins, including the structural proteins, and spike (S) [3,4]. The S gene encodes the S1 and S2 subunits, and the S1 subunit contains an N-terminal domain and a receptor-binding domain, the latter of which is associated with human infections [5]. Across its genome, the virus accumulates mutations that are associated with its transmissibility, virulence, or resistance to some neutralizing antibodies [6,7].

SARS-CoV-2 variants have been recently identified, raising concerns abouta subsequent wave of the pandemic. Since the emergence of multiple variants, the WHO and the Centers for Disease Control and Prevention (CDC) have set up a classification scheme for monitoring the potential impact of emerging variants. The variants are classified into variants of interest (VOIs), variants of concern (VOCs), and variants of high consequence (VOHCs) [8,9]. Currently, VOIs, including eta (B.1.525), iota (B.1.526), kappa (B.1.617.1), unlabeled (B.1.617.3), and epsilon (B.1.427 and B.1.429) [10], have been associated with changes in receptor binding, reduced neutralization by antibodies generated against previous infection or vaccination, reduced efficacy of treatments, potential diagnostic effects, or a predicted increase in transmissibility or disease severity. VOCs, including alpha (B.1.1.7), beta (B.1.351), delta (B.1.617.2), and gamma (P.1) [10], have been associated with an increase in transmissibility and disease severity, a significant reduction in neutralization by antibodies generated during previous infection or vaccination, reduced effectiveness of treatments or vaccines, or diagnostic detection failures. The D614G substitution is the most prevalent mutation observed [11,12]. However, VOHC-related lineages have not yet been classified [10].

These lineages have been analyzed using next generation sequencing (NGS) methods and classified using Phylogenetic Assignment of Named Global Outbreak (PANGO) lineages [13]. The use of NGS has been very useful for obtaining accurate information on genetic variability and transmission [14]. However, as outbreaks occur sporadically and cannot be predicted, it is not always possible to have all resources required to perform the tests necessary to detect SARS-CoV-2 variants, especially in resource-limited settings [15]. To overcome this limitation, both PCR and Sanger sequencing have been applied [16,17].

Here, we aimed to accurately and rapidly detect nine key S mutations (ΔH69/V70, T95I, G142D, ΔY144, K417T/N, L452R, E484K/Q, N501Y, and H655Y) in strains classified as VOCs and/or VOIs using our laboratory-developed five SARS-CoV-2 S gene (5-SSG) primers via PCR assay in conjunction with Sanger sequencing. In addition, we compared the results of our assay with those of a commercially available NGS assay to evaluate its accuracy and reliability in detecting and identifying variants.

## Materials and methods

### Primer design

The 5-SSG primer assay was designed based on the Wuhan-CoV reference sequence (Wuhan-Hu-1, NCBI accession number NC_045512.2) [18,19]. Primers were modified from the Global Initiative on Sharing Avian Influenza Data (GISAID) database, with a frequency cut-off > 1%, applied with degenerative or inosine to optimize the melting temperature (Tm), avoid repetitive sequences, and include GC content > 65%, using Gene Runner (ver. 6.0) [20,21]. NCBI-Basic Local Alignment Search Tool (NCBI-BLAST) was used to optimize the specificity for SARS-CoV-2 [22]. Sequences were also screened based on alignments using the GISAID database for species selectivity. After these assessments, five targets were selected for validation using the S mutant assay, and M13 universal sequence primers were tagged for Sanger sequencing. The sequences of the 5-SSG primers (69S forward primer, TGTAAAACGACGGCCAGTATTACCCTGACAAAGTTTTCAGATC; 69S reverse primer, CAGGAAACAGCTATGACGCGTTATTAACAATAAGTAGGGAC; 144S forward primer, TGTAAAACGACGGCCAGTCCACTGAGAAGTYTAACATAAT AAGAG; 144S reverse primer, CAGGAAACAGCTATGACTCACCAGGAGTCAAATA ACTTCTAT; 417S forward primer, TGTAAAACGACGGCCAGTGCTTTAGAACCATT GGTAGATTTG; 417S reverse primer, CAGGAAACAGCTATGACGTTTGAGATTAG ACTTCCTAAACAATC; 484S forward primer, TGTAAAACGACGGCCAGTTCTAAYA AICTTGATTCTAAGGTTG; 484S reverse primer, CAGGAAACAGCTATGACCKCCT GTGCCTGTTAAACCATT; 570S forward primer, TGTAAAACGACGGCCAGTGAAC TTCTACATGCACCAGCAAC; 570S reverse primer, CAGGAAACAGCTATGACCTG CATTCAGTTGAATCACCAC) are presented in Table 1. The 5-SSG primer-specific target mutants and lineages are summarized in S1 Table.

**Table 1:** Oligonucleotide primers used for one step PCR to detect S mutations.

Primer	Type	Start	End	Sequences (5′-3′)	Tm (°C)	Size (bp)
69S	F. primer	21672	21696	TGTAAAACGACGGCCAGT**ATTACCCTGACAAAGTTTTCAGATC**	65.8	294
	R. primer	21907	21930	CAGGAAACAGCTATGAC**GCGTTATTAACAATAAGTAGGGAC**	62.8	
144S	F. primer	21843	21869	TGTAAAACGACGGCCAGT**CCACTGAGAAGTYTAACATAATAAGAG**	63.8	513
	R. primer	22295	22320	CAGGAAACAGCTATGAC**TCACCAGGAGTCAAATAACTTCTAT**	64.1	
417S	F. primer	22226	22249	TGTAAAACGACGGCCAGT**GCTTTAGAACCATTGGTAGATTTG**	65.2	759
	R. primer	22923	22949	CAGGAAACAGCTATGAC**GTTTGAGATTAGACTTCCTAAACAATC**	65.1	
484S	F. primer	22874	22898	TGTAAAACGACGGCCAGT**TCTAAYAAICTTGATTCTAAGGTTG**	62.8	375
	R. primer	23192	23213	CAGGAAACAGCTATGAC**CKCCTGTGCCTGTTAAACCATT**	66.3	
570S	F. primer	23108	23130	TGTAAAACGACGGCCAGT**GAACTTCTACATGCACCAGCAAC**	66.2	739
	R. primer	23790	23811	CAGGAAACAGCTATGAC**CTGCATTCAGTTGAATCACCAC**	64.5	

Primers for specific target mutations in Wuhan-Hu-1-CoV were designed and conserved regions of the S gene are highlighted in bold. Primers were extended by tagging the 5′ side with M13 as a universal sequencing primer. Abbreviations: T_m_, melting temperature; F. primer, forward primer; R. primer, reverse primer; Δ, deletion; Y, C or T; K, G or T; I, inosine.

### Strain information and cultivation

To determine analytical specificity, 67 strains of viruses, bacteria, and fungi were used with or without respiratory pathogens, including 42 strains of virus (24 strains of SARS-CoV-2, Coronavirus OC43 and 229E, and 18 other viruses), 19 strains of bacteria, and six strains of fungi (Tables 2 and S2). The powdered nucleic acid of all strains used in this study was obtained from the following suppliers: Twist Bioscience (San Francisco, CA, USA), National Culture Collection for Pathogens (NCCP; Cheongju, Republic of Korea), American Type Culture Collection (ATCC; Manassas, VA, USA), Zeptometrix (Buffalo, NY, USA), Korea Bank for Pathogen Viruses (KBPV; Seoul, Republic of Korea), National Institute for Biological Standards and Control (NIBSC; Potters Bar, United Kingdom), Korean Collection for Type Cultures (KCTC; Jeongeup, Republic of Korea), and Korean Culture Center of Microorganisms (KCCM; Seoul, Republic of Korea). Other detailed strain information, including lineages and CDC classification, is shown in Table 2.

**Table 2:** PCR results and lineage information associated with the respiratory pathogens used in this study.

Group	Strain	Source	Lineage	CDC classification	PCR result
Virus	SARS-CoV-2	Twistbio-601443	alpha (B.1.1.7)	VOC	Positive
	SARS-CoV-2	Twistbio-678597	beta (B.1.351)	VOC	Positive
	SARS-CoV-2	Twistbio-710528	alpha (B.1.1.7)	VOC	Positive
	SARS-CoV-2	Twistbio-79683	gamma (P.1)	VOC	Positive
	SARS-CoV-2	NCCP-43381	alpha (B.1.1.7)	VOC	Positive
	SARS-CoV-2	NCCP-43382	beta (B.1.351)	VOC	Positive
	SARS-CoV-2	NCCP-43390	delta (B.1.617.2)	VOC	Positive
	SARS-CoV-2	NCCP-43384	epsilon (B.1.427)	VOI	Positive
	SARS-CoV-2	NCCP-43385	epsilon (B.1.429)	VOI	Positive
	SARS-CoV-2	NCCP-43386	eta (B.1.525)	VOI	Positive
	SARS-CoV-2	NCCP-43387	iota (B.1.526)	VOI	Positive
	SARS-CoV-2	NCCP-43389	kappa (B.1.617.1)	VOI	Positive
	SARS-CoV-2	NCCP-43383	zeta (P.2)	Not classified	Positive
	SARS-CoV-2	NCCP-43330	Not provided	Not classified	Positive
	SARS-CoV-2	NCCP-43331	Not provided	Not classified	Positive
	SARS-CoV-2	NCCP-43342	Not provided	Not classified	Positive
	SARS-CoV-2	NCCP-43343	Not provided	Not classified	Positive
	SARS-CoV-2	NCCP-43344	Not provided	Not classified	Positive
	SARS-CoV-2	NCCP-43345	Not provided	Not classified	Positive
	SARS-CoV-2	Zeptometrix-0810587CFHI	Not provided	Not classified	Positive
	SARS-CoV-2	Zeptometrix-0810589CFHI	Not provided	Not classified	Positive
	SARS-CoV-2	Zeptometrix-0810590CFHI	Not provided	Not classified	Positive
	Coronavirus OC43	ATCC VR1558	Not provided	Not classified	Negative
	Coronavirus 229E	ATCC-VR 740	Not provided	Not classified	Negative
	Influenza A virus	ATCC VR-810	Not provided	Not classified	Negative
	Influenza B virus	ATCC VR-1735	Not provided	Not classified	Negative
	Influenza A H1N1	ATCC VR-1683	Not provided	Not classified	Negative
	Influenza A H3N2	ATCC VR-822	Not provided	Not classified	Negative
	Influenza A H1N1	ATCC VR-219	Not provided	Not classified	Negative
	Influenza A H3N2	ATCC VR-547	Not provided	Not classified	Negative
	Respiratory syncytial virus A	ATCC VR-26	Not provided	Not classified	Negative
	Respiratory syncytial virus B	ATCC VR-955	Not provided	Not classified	Negative
	Parainfluenza type 1	ATCC VR-1380	Not provided	Not classified	Negative
Bacteria	*Staphylococcus aureus*	ATCC-29213	Not provided	Not classified	Negative
	*Streptococcus pneumoniae*	ATCC-49619	Not provided	Not classified	Negative
	*Streptococcus pyogenes*	ATCC-19615	Not provided	Not classified	Negative
	*Pseudomonas aeruginosa*	ATCC-27853	Not provided	Not classified	Negative
	*Enterobacter aerogenes*	ATCC-13048	Not provided	Not classified	Negative
	*Enterobacter cloacae*	ATCC-13047	Not provided	Not classified	Negative
	*Corynebacterium spp*.	ATCC-51860	Not provided	Not classified	Negative
	*Moraxella catarrhalis*	KCCM-42706	Not provided	Not classified	Negative
	*Haemophilus influenzae*	ATCC-9007	Not provided	Not classified	Negative
Fungi	*Aspergillus fumigatus*	Zeptometrix-Z014	Not provided	Not classified	Negative
	*Aspergillus flavus*	Zeptometrix-Z013	Not provided	Not classified	Negative
	*Aspergillus niger*	Zeptometrix-Z105	Not provided	Not classified	Negative
	*Aspergillus terreus*	Zeptometrix-Z016	Not provided	Not classified	Negative
	*Aspergillus nidulans*	ATCC-38163	Not provided	Not classified	Negative
	*Aspergillus versicolor*	ATCC-11730	Not provided	Not classified	Negative

Strains selected for assay validation (22 strains of SARS-CoV-2, 11 strains of other virus, 9 strains of bacteria, and 6 strains of fungi). Strain information, provided by the company from which the strain was acquired, is shown. Abbreviations: SARS-CoV-2, Severe acute respiratory syndrome-related coronavirus 2; Twistbio, Twist Bioscience; NCCP, National Culture Collection for Pathogens; ATCC, American Type Culture Collection; KCCM; Korean Culture Center of Microorganisms; VOC, variants of concern; VOI, variants of interest; CDC, Centers for Disease Control and Prevention.

### Clinical specimen collection and storage

As part of the routine procedure using the Allplex™ SARS-CoV-2 assay for SARS-CoV-2 testing (Seegene Inc., Seoul, Republic of Korea), anonymized residual of 17 SARS-CoV-2 positive nasopharyngeal swab specimens of patients diagnosed with SARS-CoV-2 positive between February and June 2021 were obtained and used for this study. All samples were processed using an automated nucleic acid extraction system, namely MagNA Pure 96 (Roche, Basel, Switzerland), according to the manufacturer’s protocol, and stored at −80°C until use [23].

### Analytical performance of the 5-SSG primer assay

The comparative limit of detection (LOD) of the 5-SSG primer assay was determined using heat-inactivated cultural fluids of SARS-CoV-2 (Zeptometrix-0810589CFHI) as a positive control, following the manufacturer’s instructions. Each control was 10-fold serially diluted to approximately 1.4 × 10^3^, 1.4 × 10^2^, 1.4 × 10^1^, 1.4 × 10^0^, 1.4 × 10^−1^, and 1.4 × 10^−2^ TCID_50_ (median tissue culture infectious dose)/mL. For the performance analysis of 5-SSG primers, 25 replicates were performed. The comparative LOD was determined as the minimum detectable concentration. Probit regression was used to estimate positive values with 95% confidence intervals [24].

### One-step RT-PCR and agarose gel electrophoresis

The template (2.5 ng) from viral, bacterial, and fungal strains was added for one-step RT-PCR (Nanohelix Co., Daejeon, Republic of Korea) analysis, which was performed using a SeeAmp (Seegene) instrument. PCR assays with the 5-SSG primers were performed using the following thermal cycling conditions: 45°C for 15 min (reverse transcription), followed by 94°C for 15 min (initial denaturation), and 45 cycles of 94°C for 10 s (denaturation), 64°C for 30 s (annealing), and 72°C for 30 s (extension). A final extension step was conducted at 72°C for 5 min. Next, the PCR products were analyzed using 2% agarose gel electrophoresis with 0.5× TBE buffer, and the gels were stained with ethidium bromide (Biosesang, Seongnam, Republic of Korea). PCR amplicons from the 67 samples were analyzed using agarose gel electrophoresis in a horizontal unit (CBS Scientific, San Diego, CA, USA) operating at 280 V for 28 min, and the band sizes on ethidium bromide-stained gels were quantified using a Gel-Doc XR+ system (Bio-Rad Laboratories, Hercules, CA, USA).

### PCR product purification and sequence analysis

All PCR-positive products were purified with MEGAquick-spin™ plus (iNtRON Biotechnology, Seongnam, Republic of Korea), according to the manufacturer’s instructions [25]. The sequence analysis of PCR products (partial S gene amplified to ~800 bp) was performed using the 5-SSG primers (5′ tagged M13 primer) and the BigDye Terminator v3.1 cycle sequencing kit reagent (Applied Biosystems, Foster City, CA, USA). The sequence analysis conditions were as follows: 96°C for 1 min (incubation), followed by 25 cycles of 96°C for 10 s (denaturation), 50°C for 5 s (annealing), and 60°C for 4 min (extension). Dye-labeled products were analyzed using an ABI 3730 sequencer (Applied Biosystems). Sequencing chromatograms were analyzed manually using Variant Reporter™ v3.0 software (Applied Biosystems). Samples were classified as mutants if the sequencing results from the specific regions matched those of lineage information [26].

### NGS and data analysis

NGS was performed using the SARS-CoV-2 FLEX Panels (Paragon Genomics, Hayward, CA, USA) and an Illumina MiSeq platform (Illumina, San Diego, CA, USA) in accordance with the manufacturer’s instructions [27]. Reverse transcription was performed using 55 ng of nucleic acid, and multiplex PCR was performed using 343 pairs of primers. A second PCR was conducted using CleanPlex Dual-Indexed PCR Primers for Illumina® Set A (Paragon Genomics). The final library was sequenced on an Illumina MiSeq platform (Illumina) with 2 × 150bp flow cells using a MiSeq Micro Reagent Kit v2 (300 cycles).

Next, NGS assays were analyzed using the Flomics pipeline (Flomics, Barcelona, Spain). The processing pipeline comprised FastQC v0.11.9 (quality control), followed by fastp v0.20.1. (adapter trimming), Bowtie2 (reference alignment), and iVar v1.2.2. (variant calling). The viral lineage was accessed using the GISAID database, and PANGO Lineages [28]. In NGS analysis, depths of less than 10× were identified by read-depth segmentation in an integrated genomics viewer [27].

### Ethics statement

Ethical aspects of this study were reviewed and approved by the Seegene Medical Foundation Institutional Review Board (approval number, SMF-IRB-2021-006), provided that after conducting the laboratory diagnoses of SARS-CoV-2 testing, the remaining samples be destroyed. All data were fully anonymized administrative data without patient identifiers, and patient consent was waived by the institutional review board.

## Results

### Optimization of five SARS-CoV-2 primer pairs for S mutants

**Fig 1:**
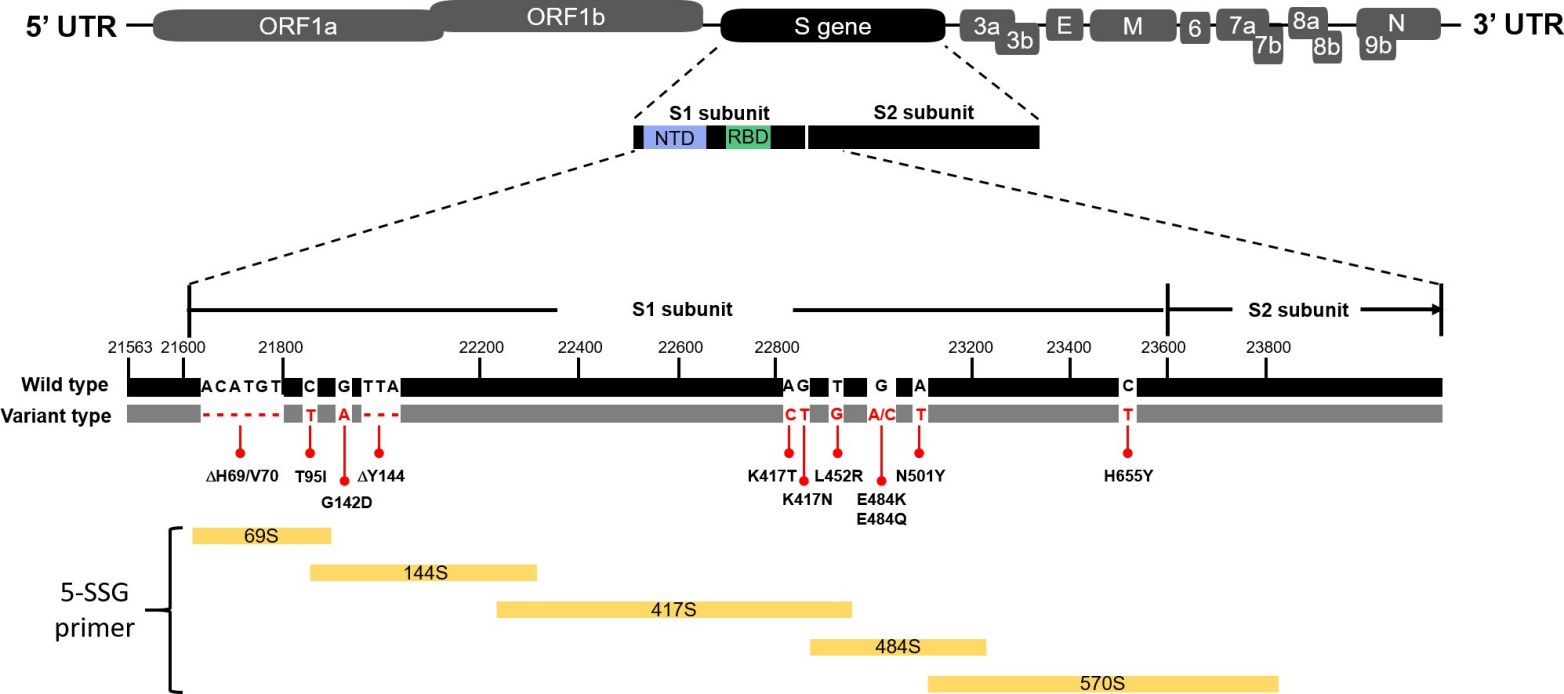
Overall schematic structures of SARS-CoV-2 spike gene and derived 5-SSG primers.

The 5-SSG primers consisted of five primer pairs, including 69S, 144S, 417S, 484S, and 570S. The 69S primer pair for 4 mutants (A67V, ΔH69/V70, D80A, and T95I), 144S primer pair for 9 mutants (D138Y, G142D, ΔY144, W152C, E154K, ΔE156/F157, R158G, R190S, and D215G), 417S primer pair for 4 mutants (D253G, K417T, K417N, and L452R), 484S primer pair for 4 mutants (T478K, E484K, E484Q, and N501Y), and 570S primer pair for 8 mutants (A570D, D614G, H655Y, Q677H, P681H, P681R, A701V, and T716I) were designed to detect target mutants (Table 1). The lineage and CDC classification information of each primer are shown in S1 Table. All target mutants were efficiently included in S gene coverage (Table 1, Fig 1, and S1 Table).

### PCR efficiency and 5-SSG primers performance analysis

The analytical performance of the 5-SSG primers was confirmed using a total of 67 strains, including viruses, bacteria, and fungi. The PCR results were determined to be positive or negative based on the expected PCR product sizes (Tables 1, 2 and S2). As shown in Tables 2 and S2, the 5-SSG primer pairs achieved consistent results for twenty-two strains of SARS-CoV-2, whereas a negative result was obtained for the remaining 45 stains (other viruses, bacteria, and fungi).

### Determination of the analytical sensitivity of the 5-SSG primers

Analytical sensitivity, positivity rate, and LOD were estimated using 25 replicates of positive strains (Zeptometrix-0810590CFHI) at six different concentrations, from approximately 1.4 × 10^−2^ to 1.4 × 10^3^ TCID_50_/mL (Table 3). Results (probit analysis) showed that the 95% LOD was approximately 3.7 × 10^1^ TCID_50_/mL for 69S, 9.8 × 10^1^ TCID_50_/mL for 144S, 6.6 × 10^1^ TCID_50_/mL for 417S, 3.9 × 10^1^ TCID_50_/mL for 484S, and 7.2 × 10^1^ TCID_50_/mL for 570S (Table 3). Assay results showed 100% reproducibility for all 5-SSG primer pairs, even for concentrations of as low as approximately 1.4 × 10^2^ TCID_50_/mL. The LOD was approximately 1.4 × 10^1^ TCID_50_/mL, except for in the 69S and 484S assays, which were 10 times more sensitive than the 144S, 417S, and 570S assays.

**Table 3:** Evaluation of detection limit in target regions.

Primer pair	Conc. (TCID_50_/mL)	Reactions	Positive	Positive rate (%)	LOD 95% level (TCID_50_/mL)
69S	1.4 × 10^3^	25	25	100	3.7 × 10^1^
	1.4 × 10^2^	25	25	100	
	1.4 × 10^1^	25	20	80	
	1.4 × 10^0^	25	7	28	
	1.4 × 10^−1^	25	0	0	
	1.4 × 10^−2^	25	0	0	
144S	1.4 × 10^3^	25	25	100	9.8 × 10^1^
	1.4 × 10^2^	25	25	100	
	1.4 × 10^1^	25	4	16	
	1.4 × 10^0^	25	0	0	
	1.4 × 10^−1^	25	0	0	
	1.4 × 10^−2^	25	0	0	
417S	1.4 × 10^3^	25	25	100	6.6 × 10^1^
	1.4 × 10^2^	25	25	100	
	1.4 × 10^1^	25	8	32	
	1.4 × 10^0^	25	0	0	
	1.4 × 10^−1^	25	0	0	
	1.4 × 10^−2^	25	0	0	
484S	1.4 × 10^3^	25	25	100	3.9 × 10^1^
	1.4 × 10^2^	25	25	100	
	1.4 × 10^1^	25	18	72	
	1.4 × 10^0^	25	1	4	
	1.4 × 10^−1^	25	0	0	
	1.4 × 10^−2^	25	0	0	
570S	1.4 × 10^3^	25	25	100	7.2 × 10^1^
	1.4 × 10^2^	25	25	100	
	1.4 × 10^1^	25	7	28	
	1.4 × 10^0^	25	0	0	
	1.4 × 10^−1^	25	0	0	
	1.4 × 10^−2^	25	0	0	

The 5-SSG primer-PCR reactions performed using ten-fold diluted positive samples. The LOD 95% data were estimated using the probit regression analysis. Abbreviations: Conc., concentration; TCID_50_, median tissue culture infective dose; LOD, limit of detection.

### Sanger sequencing analysis

**Fig 2:**
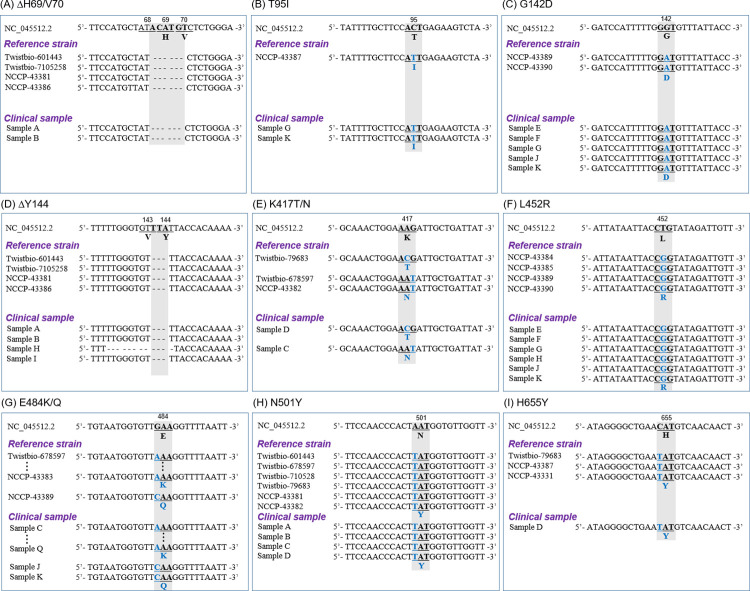
Sequence analysis of SARS-CoV-2 S protein. (A) ΔH69/V70, and (B) T95I from 69S; (C) G142D, and (D) ΔY144 from 144S; (E) K417T/N, and (F) L452R from 417S; (G) E484K/Q, and (H) N501Y from 484S; (I) H655Y from 570S. Sequences showing deletions or conversions are highlighted for comparison with the Wuhan-Hu-1-CoV sequence.

As shown in Table 2, the nucleotide sequences of the positive PCR products obtained from 22 strains were compared with the existing S mutations through the Sanger Sequencing method using the M13 primer. The key deletion mutations ΔH69/V70 and ΔY144 were found in four strains (Twistbio-601443, Twistbio-7105258, NCCP-43381, and NCCP-43386). In addition, other substitution mutations were found to be 100% consistent with those in each strain’s corresponding lineage, except for two cases in which substitutions at T95I for the NCCP-43390 strain and W152C for the NCCP-43384 strain were mismatched in the CDC classification (Figs 2 and S1, and S1 Table). Overall, it was confirmed through Sanger sequencing that the 5-SSG primers can detect predominant S gene mutations of SARS-CoV-2 observed in the major mutant strain categories, VOIs and VOCs, with high sensitivity and efficiency.

### Comparison of mutants detected by 5-SSG primer assay using Sanger sequencing versus NGS

SARS-CoV-2 mutants have been genetically characterized using NGS-based lineages [13,29]. To confirm the detection accuracy of the 5-SSG primer assay developed in this study for the SARS-CoV-2 mutants, NGS analysis results were used for a comparison. The NGS assay identified three strains (Twistbio-710528, Twistbio-601443, and NCCP-43381) as B.1.1.7, two (Twistbio-678597 and NCCP-43382) as B.1.351, one (Twistbio-79683) as P.1, one (NCCP-43390) as B.1.617.2, one (NCCP-43384) as B.1.427, one (NCCP-43385) as B.1.429, one (NCCP-43386) as B.1.525, one (NCCP-43387) as B.1.526, and one (NCCP-43389) as B.1.617.1. The remaining ten strains (NCCP-43330, NCCP-43331, NCCP-43342, NCCP-43343, NCCP-43344, NCCP-43345, NCCP-43383, Zeptometrix-0810587CFHI, Zeptometrix-0810589CFHI, and Zeptometrix-0810589CFHI) were genetically classified into another lineage (Table 4). Results of NGS and Sanger sequencing using the 5-SSG primers showed 100% consistency for all strains, including T95I for the NCCP-43390 strain and W152C for the NCCP-43384 strain (Table 4). Taken together, the 5-SSG primer assay is very efficient in detecting SARS-CoV-2 major S gene mutant strains.

**Table 4:** Comparison of 5-SSG primers target mutations sequence of Sanger sequencing and NGS.

Grades of concern	Lineage	Source	Sequence analysis method		Final Determination
			NGS	Sanger sequencing	
VOC	B.1.1.7	Twistbio-710528	ΔH69/V70, ΔY144, N501Y, A570D, D614G, P681H, T716I	ΔH69/V70, ΔY144, N501Y, A570D, D614G, P681H, T716I	Match
		Twistbio-601443	ΔH69/V70, ΔY144, N501Y, A570D, D614G, P681H, T716I	ΔH69/V70, ΔY144, N501Y, A570D, D614G, P681H, T716I	Match
		NCCP-43381	ΔH69/V70, ΔY144, N501Y, A570D, D614G, P681H, R682Q, T716I	ΔH69/V70, ΔY144, N501Y, A570D, D614G, P681H, R682Q, T716I	Match
	B.1.351	Twistbio-678597	D80A, D215G, ΔLAL242-244, K417N, E484K, N501Y, D614G, A701V	D80A, D215G, ΔLAL242-244, K417N, E484K, N501Y, D614G, A701V	Match
		NCCP-43382	L54F, D80A, D215G, ΔLAL242-244, K417N, E484K, N501Y, D614G, A701V	L54F, D80A, D215G, ΔLAL242-244, K417N, E484K, N501Y, D614G, A701V	Match
	P.1	Twistbio-79683	D138Y, R190S, K417T, E484K, N501Y, D614G, H655Y	D138Y, R190S, K417T, E484K, N501Y, D614G, H655Y	Match
	B.1.617.2	NCCP-43390	G142D, ΔE156/F157, R158G, L452R, T478K, Q613H, D614G, P681R, R682W	G142D, ΔE156/F157, R158G, L452R, T478K, Q613H, D614G, P681R, R682W	Match
VOI	B.1.427	NCCP-43384	W152C, L452R, D614G	W152C, L452R, D614G	Match
	B.1.429	NCCP-43385	W152C, L452R, D614G	W152C, L452R, D614G	Match
	B.1.525	NCCP-43386	Q52R, A67V, ΔH69/V70, ΔY144, E484K, D614G, Q677H	Q52R, A67V, ΔH69/V70, ΔY144, E484K, D614G, Q677H	Match
	B.1.526	NCCP-43387	T95I, D253G, E484K, D614G, H655Y, A701V	T95I, D253G, E484K, D614G, H655Y, A701V	Match
	B.1.617.1	NCCP-43389	G142D, E154K, L452R, E484Q, D614G, P681R, R682Q	G142D, E154K, L452R, E484Q, D614G, P681R, R682Q	Match
Not included	P.2	NCCP-43383	E484K, D614G	E484K, D614G	Match
	B	NCCP-43330	-	-	Match
	A	NCCP-43331	H655Y	H655Y	Match
	B	NCCP-43342	-	-	Match
	B.1.1-	NCCP-43343	D614G, R682Q	D614G, R682Q	Match
	B.1-	NCCP-43344	D215H, D614G, R682Q	D215H, D614G, R682Q	Match
	B.1.497	NCCP-43345	D614G, ΔQTQTN675-679, R682L	D614G, ΔQTQTN675-679, R682L	Match
	A	Zeptometrix -0810587CFHI	D215/L216insKLRS, ΔQTQTN675-679	D215/L216insKLRS, ΔQTQTN675-679	Match
	B	Zeptometrix -0810589CFHI	N74K, S247R, ΔNSPRRARSVA679-688	N74K, S247R, ΔNSPRRARSVA679-688	Match
	A	Zeptometrix -0810590CFHI	S247R, V367F, R682Q,	S247R, V367F, R682Q	Match

Abbreviations: VOC, Variant of concern; VOI, variant of interest; Δ, deletion. Low-coverage NGS data are marked in underline.

### Validation of clinical sample variants using the 5-SSG primers

To confirm the detection accuracy of the 5-SSG primer assay using clinical samples, Sanger sequencing results were compared with those of NGS analysis (Table 5). The results of VOCs (B.1.1.7, B.1.351, P.1, and B.1.617.2), VOIs (B.1.429, B.1.525, and B.1.617.1), and the remaining two lineages (B.1.497, B.1.619) were compared (Table 5). NGS assays and Sanger sequencing using the 5-SSG primers showed 100% consistent results for all strains. We concluded that the 5-SSG primer assay also had a very efficient performance with clinical samples.

**Table 5:** Validation of 5-SSG primers target mutations sequence using clinical samples.

Grades of concern	Lineage	Sample	Sequence analysis method		Final Determination
			NGS	Sanger sequencing	
VOC	B.1.1.7	Sample A	ΔH69/V70, ΔY144, N501Y, A570D, D614G, P681H, T716I	ΔH69/V70, ΔY144, N501Y, A570D, D614G, P681H, T716I	Match
		Sample B	ΔH69/V70, ΔY144, N501Y, A570D, D614G, P681H, T716I	ΔH69/V70, ΔY144, N501Y, A570D, D614G, P681H, T716I	Match
	B.1.351	Sample C	D80A, D215G, ΔLAL242-244, K417N, E484K, N501Y, D614G, A701V	D80A, D215G, ΔLAL242-244, K417N, E484K, N501Y, D614G, A701V	Match
	P.1	Sample D	D138Y, R190S, K417T, E484K, N501Y, D614G, H655Y	D138Y, R190S, K417T, E484K, N501Y, D614G, H655Y	Match
	B.1.617.2	Sample E	G142D, ΔE156/F157, R158G, L452R, T478K, D614G, P681R	G142D, ΔE156/F157, R158G, L452R, T478K, D614G, P681R	Match
		Sample F	G142D, ΔE156/F157, R158G, L452R, T478K, D614G, P681R	G142D, ΔE156/F157, R158G, L452R, T478K, D614G, P681R	Match
		Sample G	T95I, G142D, ΔE156/F157, R158G, L452R, T478K, D614G, P681R	T95I, G142D, ΔE156/F157, R158G, L452R, T478K, D614G, P681R	Match
VOI	B.1.429	Sample H	ΔLGVY141-144, W152C, G252V, S256L, L452R, D614G	ΔLGVY141-144, W152C, G252V, S256L, L452R, D614G	Match
	B.1.525	Sample I	Q52R, A67V, ΔH69/V70, ΔY144, E484K, D614G, Q677H	Q52R, A67V, ΔH69/V70, ΔY144, E484K, D614G, Q677H	Match
	B.1.617.1	Sample J	G142D, E154K, L452R, E484Q, D614G, P681R	G142D, E154K, L452R, E484Q, D614G, P681R	Match
		Sample K	T95I, G142D, E154K, L452R, E484Q, D614G, P681R	T95I, G142D, E154K, L452R, E484Q, D614G, P681R	Match
Not included	B.1.497	Sample L	D614G	D614G	Match
		Sample M	D614G	D614G	Match
		Sample N	D614G	D614G	Match
	B.1.619	Sample O	I210T, N440K, E484K, D614G	I210T, N440K, E484K, D614G	Match
		Sample P	I210T, N440K, E484K, D614G	I210T, N440K, E484K, D614G	Match
		Sample Q	I210T, N440K, E484K, D614G	I210T, N440K, E484K, D614G	Match

Abbreviations: VOC, Variant of concern; VOI, variant of interest; Δ, deletion. Low-coverage NGS data are marked in underline.

## Discussion

In the ongoing COVID-19 pandemic, it has been demonstrated that the rapid detection of the pathogen is critical to prevent the rampant spread of the disease [30]. The emergence of SARS-CoV-2 variants, which are associated with increased transmission, disease severity, and resistance to vaccines, is a grave concern [31]. The alpha (B.1.1.7) and beta (B.1.351) lineages of SARS-CoV-2, which account for 98.7% of total variant cases, contain the mutations ΔH69/V70, E484K, and N501Y [32]. S protein-based vaccines might provide less protection against these mutants (ΔH69/V70, E484K, and N501Y) of SARS-CoV-2 [33]. Therefore, a simple and rapid screening assay to monitor the emergence and spread of these variants is essential to implement public health strategies [31].

In this study, we developed primers for the rapid and accurate detection of the key mutants of the S gene of SARS-CoV-2 and evaluated the reliability and reproducibility of these primers (Tables 2 and 3). The 5-SSG primers (69S, 144S, 417S, 484S, and 570S) had high analytical specificity for SARS-CoV-2 strains and no cross-reactivity with other strains (Tables 2 and S2). Results of Sanger sequencing using 5-SSG primers and commercial NGS were in 100% agreement; however, the three approaches differed in their ability to detect the E484K and D215G variants of the beta (B.1.351) lineage, E484K of the gamma (P.1) lineage, and G142D of the delta (B.1.617.2) lineage (Tables 4 and 5). These results indicate that in NGS analysis, low-depth levels of mutants (G142D, D215G, and E484K) are detected, because the target amplification is affected by a mutation in the reverse primer binding site (ΔE156/F157, R158G, ΔLAL242-244, and N501Y). In addition, NGS is limited to the environment in which the equipment is built, and it also takes a longer as it is more complex than typical Sanger sequencing [34]. Therefore, the Sanger sequencing-based 5-SSG primer assay system can rapidly and accurately detect key mutants of the S gene without resource constraint, and is a useful tool that can overcome the limitation of relatively low read-depth caused by mutations in primer-binding site during NGS analysis.

One limitation of this study is that the performance of the 5-SSG primers was tested using small numbers of clinical samples through Sanger sequencing and NGS analysis, and thus, further studies using a larger number of clinical samples should be performed. In addition, the current 5-SSG primer system can identify lambda (C.37) variants with the 417S primer set, but the ΔRSYLTPGD246-253N mutation affects the 144S reverse primer. Therefore, improvements in primer performance for detection of additional variants (e.g. B. 1.617.3 and B. 1.621) and the development of new primers should be pursued in future studies.

Collectively, the 5-SSG primer assay system has high PCR sensitivity specifically for SARS-CoV-2 and is a useful tool that can detect various S gene mutants very quickly and accurately, thereby contributing to the faster control of pathogen transmission in the population.

## Supporting information

S1 Fig Sequence chromatograms of raw data for SARS-CoV-2 S protein. (A) ΔH69/V70 and (B) T95I from 69S; (C) G142D and (D) ΔY144 from 144S; (E) K417T, (F) K417N, and (G) L452R from 417S; (H) E484K, (I) E484Q, and (J) N501Y from 484S; (K) H655Y from 570S. Chromatograms showing deletions or conversions are highlighted for comparison with the Wuhan-Hu-1-CoV sequence.
(TIF)

S1 Table Primers-specific target mutant and lineage classification, and Sanger sequencing result. Abbreviations: VOI, Variants of Interest; VOC, Variants of Concern; Twistbio, Twist Bioscience; NCCP, National Culture Collection for Pathogens.
(PDF)

S2 Table PCR results and non-respiratory pathogen strain information used in this study. Abbreviations: SARS-CoV-2, Severe acute respiratory syndrome-related coronavirus 2; ATCC, American Type Culture Collection; KBPV, Korea Bank for Pathogen Viruses; NIBSC, National Institute for Biological Standards and Control; KCTC, Korean Collection for Type Cultures.
(PDF)

S1 Raw data (ZIP)

## References

[1] Gisondi P, S PI, Bordin C, Alaibac M, Girolomoni G, Naldi L. Cutaneous manifestations of SARS-CoV-2 infection: a clinical update. J Eur Acad Dermatol Venereol. 2020;34(11):2499–504. Epub 2020/06/26. doi: 10.1111/jdv.16774 ; PubMed Central PMCID: PMC7362144. 32585074 PMC7362144

[2] Adhikari SP, Meng S, Wu YJ, Mao YP, Ye RX, Wang QZ, et al. Epidemiology, causes, clinical manifestation and diagnosis, prevention and control of coronavirus disease (COVID-19) during the early outbreak period: a scoping review. Infect Dis Poverty. 2020;9(1):29. Epub 2020/03/19. doi: 10.1186/s40249-020-00646-x ; PubMed Central PMCID: PMC7079521. 32183901 PMC7079521

[3] Sacar Demirci MD, Adan A. Computational analysis of microRNA-mediated interactions in SARS-CoV-2 infection. PeerJ. 2020;8:e9369. Epub 2020/06/18. doi: 10.7717/peerj.9369 ; PubMed Central PMCID: PMC7278893. 32547891 PMC7278893

[4] Khailany RA, Safdar M, Ozaslan M. Genomic characterization of a novel SARS-CoV-2. Gene Rep. 2020;19:100682. Epub 2020/04/18. doi: 10.1016/j.genrep.2020.100682 ; PubMed Central PMCID: PMC7161481. 32300673 PMC7161481

[5] Huang Y, Yang C, Xu XF, Xu W, Liu SW. Structural and functional properties of SARS-CoV-2 spike protein: potential antivirus drug development for COVID-19. Acta Pharmacol Sin. 2020;41(9):1141–9. Epub 2020/08/05. doi: 10.1038/s41401-020-0485-4 ; PubMed Central PMCID: PMC7396720. 32747721 PMC7396720

[6] Li Q, Wu J, Nie J, Zhang L, Hao H, Liu S, et al. The Impact of Mutations in SARS-CoV-2 Spike on Viral Infectivity and Antigenicity. Cell. 2020;182(5):1284–94 e9. Epub 2020/07/31. doi: 10.1016/j.cell.2020.07.012 ; PubMed Central PMCID: PMC7366990. 32730807 PMC7366990

[7] Roy C, Mandal SM, Mondal SK, Mukherjee S, Mapder T, Ghosh W, et al. Trends of mutation accumulation across global SARS-CoV-2 genomes: Implications for the evolution of the novel coronavirus. Genomics. 2020;112(6):5331–42. Epub 2020/11/09. doi: 10.1016/j.ygeno.2020.11.003 ; PubMed Central PMCID: PMC7644180. 33161087 PMC7644180

[8] Konings F, Perkins MD, Kuhn JH, Pallen MJ, Alm EJ, Archer BN, et al. SARS-CoV-2 Variants of Interest and Concern naming scheme conducive for global discourse. Nat Microbiol. 2021;6(7):821–3. Epub 2021/06/11. doi: 10.1038/s41564-021-00932-w . 34108654

[9] Aleem A, Akbar Samad AB, Slenker AK. Emerging Variants of SARS-CoV-2 And Novel Therapeutics Against Coronavirus (COVID-19). StatPearls. Treasure Island (FL): StatPearls Publishing Copyright © 2021, StatPearls Publishing LLC.; 2021. 34033342

[10] Centers for Disease Control and Prevention: SARS-CoV-2 Variant Classifications and Definitions—03 August 2021. [cited 2021 03 August]. Available from: https://www.cdc.gov/coronavirus/2019-ncov/variants/variant-info.html.

[11] Korber B, Fischer WM, Gnanakaran S, Yoon H, Theiler J, Abfalterer W, et al. Tracking Changes in SARS-CoV-2 Spike: Evidence that D614G Increases Infectivity of the COVID-19 Virus. Cell. 2020;182(4):812–27 e19. Epub 2020/07/23. doi: 10.1016/j.cell.2020.06.043 ; PubMed Central PMCID: PMC7332439. 32697968 PMC7332439

[12] Yurkovetskiy L, Wang X, Pascal KE, Tomkins-Tinch C, Nyalile TP, Wang Y, et al. Structural and Functional Analysis of the D614G SARS-CoV-2 Spike Protein Variant. Cell. 2020;183(3):739–51 e8. Epub 2020/09/30. doi: 10.1016/j.cell.2020.09.032 ; PubMed Central PMCID: PMC7492024. 32991842 PMC7492024

[13] Zhou P, Yang XL, Wang XG, Hu B, Zhang L, Zhang W, et al. A pneumonia outbreak associated with a new coronavirus of probable bat origin. Nature. 2020;579(7798):270–3. Epub 2020/02/06. doi: 10.1038/s41586-020-2012-7 ; PubMed Central PMCID: PMC7095418. 32015507 PMC7095418

[14] Chiara M, D’Erchia AM, Gissi C, Manzari C, Parisi A, Resta N, et al. Next generation sequencing of SARS-CoV-2 genomes: challenges, applications and opportunities. Brief Bioinform. 2021;22(2):616–30. Epub 2020/12/07. doi: 10.1093/bib/bbaa297 ; PubMed Central PMCID: PMC7799330. 33279989 PMC7799330

[15] Pillay S, Giandhari J, Tegally H, Wilkinson E, Chimukangara B, Lessells R, et al. Whole Genome Sequencing of SARS-CoV-2: Adapting Illumina Protocols for Quick and Accurate Outbreak Investigation during a Pandemic. Genes (Basel). 2020;11(8). Epub 2020/08/23. doi: 10.3390/genes11080949 ; PubMed Central PMCID: PMC7464704. 32824573 PMC7464704

[16] Matsuda K. PCR-Based Detection Methods for Single-Nucleotide Polymorphism or Mutation: Real-Time PCR and Its Substantial Contribution Toward Technological Refinement. Adv Clin Chem. 2017;80:45–72. Epub 2017/04/23. doi: 10.1016/bs.acc.2016.11.002 . 28431642

[17] Deng J, Huang H, Yu X, Jin J, Lin W, Li F, et al. DiSNPindel: improved intra-individual SNP and InDel detection in direct amplicon sequencing of a diploid. BMC Bioinformatics. 2015;16:343. Epub 2015/10/27. doi: 10.1186/s12859-015-0790-y ; PubMed Central PMCID: PMC4619477. 26498606 PMC4619477

[18] Laskar R, Ali S. Phylo-geo-network and haplogroup analysis of 611 novel coronavirus (SARS-CoV-2) genomes from India. Life Sci Alliance. 2021;4(5). Epub 2021/03/18. doi: 10.26508/lsa.202000925 ; PubMed Central PMCID: PMC7994317. 33727249 PMC7994317

[19] Long SW, Olsen RJ, Christensen PA, Subedi S, Olson R, Davis JJ, et al. Sequence Analysis of 20,453 Severe Acute Respiratory Syndrome Coronavirus 2 Genomes from the Houston Metropolitan Area Identifies the Emergence and Widespread Distribution of Multiple Isolates of All Major Variants of Concern. Am J Pathol. 2021;191(6):983–92. Epub 2021/03/21. doi: 10.1016/j.ajpath.2021.03.004 ; PubMed Central PMCID: PMC7962948. 33741335 PMC7962948

[20] Riet J, Ramos LRV, Lewis RV, Marins LF. Improving the PCR protocol to amplify a repetitive DNA sequence. Genet Mol Res. 2017;16(3). Epub 2017/10/04. doi: 10.4238/gmr16039796 . 28973773

[21] Yamada T, Soma H, Morishita S. PrimerStation: a highly specific multiplex genomic PCR primer design server for the human genome. Nucleic Acids Res. 2006;34(Web Server issue):W665–9. Epub 2006/07/18. doi: 10.1093/nar/gkl297 ; PubMed Central PMCID: PMC1538814. 16845094 PMC1538814

[22] Johnson M, Zaretskaya I, Raytselis Y, Merezhuk Y, McGinnis S, Madden TL. NCBI BLAST: a better web interface. Nucleic Acids Res. 2008;36(Web Server issue):W5–9. Epub 2008/04/29. doi: 10.1093/nar/gkn201 ; PubMed Central PMCID: PMC2447716. 18440982 PMC2447716

[23] Mengelle C, Mansuy JM, Sandres-Saune K, Barthe C, Boineau J, Izopet J. Prospective evaluation of a new automated nucleic acid extraction system using routine clinical respiratory specimens. J Med Virol. 2012;84(6):906–11. Epub 2012/04/14. doi: 10.1002/jmv.23281 ; PubMed Central PMCID: PMC7166974. 22499014 PMC7166974

[24] Wallis RS. PROBIT: a computer program analysis. J Immunol Methods. 1991;145(1–2):267–8. Epub 1991/12/15. doi: 10.1016/0022-1759(91)90338-g . 1765663

[25] Ayerbe-Algaba R, Gil-Marques ML, Jimenez-Mejias ME, Sanchez-Encinales V, Parra-Millan R, Pachon-Ibanez ME, et al. Synergistic Activity of Niclosamide in Combination With Colistin Against Colistin-Susceptible and Colistin-Resistant Acinetobacter baumannii and Klebsiella pneumoniae. Front Cell Infect Microbiol. 2018;8:348. Epub 2018/10/20. doi: 10.3389/fcimb.2018.00348 ; PubMed Central PMCID: PMC6178895. 30338245 PMC6178895

[26] Centers for Disease Control and Prevention: SARS-CoV-2 Variant Classifications and Definitions—23 June 2021. [cited 2021 23 June]. Available from: https://www.cdc.gov/coronavirus/2019-ncov/variants/variant-info.html.

[27] Charre C, Ginevra C, Sabatier M, Regue H, Destras G, Brun S, et al. Evaluation of NGS-based approaches for SARS-CoV-2 whole genome characterisation. Virus Evol. 2020;6(2):veaa075. Epub 2020/12/16. doi: 10.1093/ve/veaa075 ; PubMed Central PMCID: PMC7665770. 33318859 PMC7665770

[28] Hirotsu Y, Omata M. Detection of R.1 lineage severe acute respiratory syndrome coronavirus 2 (SARS-CoV-2) with spike protein W152L/E484K/G769V mutations in Japan. PLoS Pathog. 2021;17(6):e1009619. Epub 2021/06/08. doi: 10.1371/journal.ppat.1009619 . 34097716 PMC8238201

[29] Ntoumi F, Mfoutou Mapanguy CC, Tomazatos A, Pallerla SR, Linh LTK, Casadei N, et al. Genomic surveillance of SARS-CoV-2 in the Republic of Congo. Int J Infect Dis. 2021;105:735–8. Epub 2021/03/20. doi: 10.1016/j.ijid.2021.03.036 ; PubMed Central PMCID: PMC7959680. 33737129 PMC7959680

[30] Ding X, Yin K, Li Z, Lalla RV, Ballesteros E, Sfeir MM, et al. Ultrasensitive and visual detection of SARS-CoV-2 using all-in-one dual CRISPR-Cas12a assay. Nat Commun. 2020;11(1):4711. Epub 2020/09/20. doi: 10.1038/s41467-020-18575-6 ; PubMed Central PMCID: PMC7501862. 32948757 PMC7501862

[31] Banada P, Green R, Banik S, Chopoorian A, Streck D, Jones R, et al. A Simple RT-PCR Melting temperature Assay to Rapidly Screen for Widely Circulating SARS-CoV-2 Variants. medRxiv. 2021. Epub 2021/03/25. doi: 10.1101/2021.03.05.21252709 ; PubMed Central PMCID: PMC7987051. 34288729 PMC8451443

[32] Public Health England. Variants: distribution of cases data [cited 2021 7 April]. Available from: https://www.gov.uk/government/publications/covid-19-variants-genomically-confirmed-case-numbers/variants-distribution-of-cases-data.

[33] Xie X, Liu Y, Liu J, Zhang X, Zou J, Fontes-Garfias CR, et al. Neutralization of SARS-CoV-2 spike 69/70 deletion, E484K and N501Y variants by BNT162b2 vaccine-elicited sera. Nat Med. 2021. Epub 2021/02/10. doi: 10.1038/s41591-021-01270-4 . 33558724

[34] Lee Y, Clark EW, Milan MSD, Champagne C, Michael KS, Awad MM, et al. Turnaround Time of Plasma Next-Generation Sequencing in Thoracic Oncology Patients: A Quality Improvement Analysis. JCO Precis Oncol. 2020;4. Epub 2020/10/06. doi: 10.1200/PO.20.00121 ; PubMed Central PMCID: PMC7529535 manuscript. 33015530 PMC7529535

