# Supplementary figures and images for: Development of an efficient Sanger sequencing-based assay for detecting SARS-CoV-2 spike mutations
Source: PLoS One. 2021 Dec 14;16(12):e0260850. doi: 10.1371/journal.pone.0260850 (PMC8670694; doi:10.1371/journal.pone.0260850)

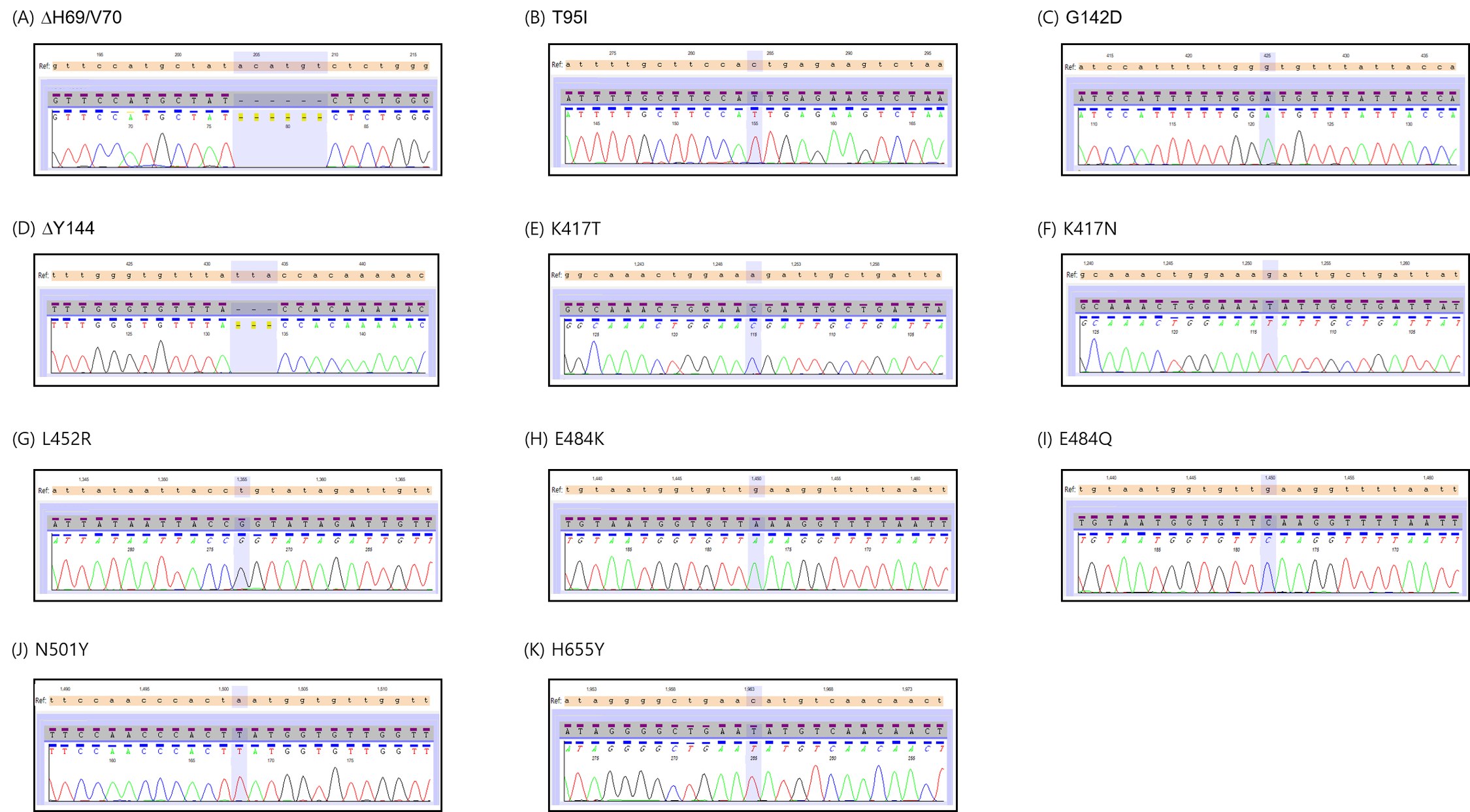

Supplement: S1 Fig — (A) ΔH69/V70 and (B) T95I from 69S; (C) G142D and (D) ΔY144 from 144S; (E) K417T, (F) K417N, and (G) L452R from 417S; (H) E484K, (I) E484Q, and (J) N501Y from 484S; (K) H655Y from 570S. Chromatograms showing deletions or conversions are highlighted for comparison with the Wuhan-Hu-1-CoV sequence. (TIF) [file pone.0260850.s001.tif]
