# Supplementary material for: Development of an efficient Sanger sequencing-based assay for detecting SARS-CoV-2 spike mutations
Source: PLoS One. 2021 Dec 14;16(12):e0260850. doi: 10.1371/journal.pone.0260850 (PMC8670694; doi:10.1371/journal.pone.0260850)
Supplement: S1 Table — Abbreviations: VOI, Variants of Interest; VOC, Variants of Concern; Twistbio, Twist Bioscience; NCCP, National Culture Collection for Pathogens. (PDF) [file pone.0260850.s002.pdf]

Primer	Target variant	CDC classification and lineage		Sanger sequencing result (detection strain)
		VOI	VOC	
69S	A67V	eta (B.1.525)		NCCP-43386
	∆H69/V70	eta (B.1.525)	alpha (B.1.1.7)	Twistbio-601443, Twistbio-7105258, NCCP-43381, NCCP-43386
	D80A		beta (B.1.351)	Twistbio-678597, NCCP-43382
	T95I	iota (B.1.526)	delta (1.617.2)	NCCP-43387
144S	D138Y		gamma (P.1)	Twistbio-79683
	G142D	kappa (1.617.1)	delta (1.617.2)	NCCP-43389, NCCP-43390
	∆Y144	eta (B.1.525)	alpha (B.1.1.7)	Twistbio-601443, Twistbio-7105258, NCCP-43381, NCCP-43386
	W152C	epsilon (B.1.429)		NCCP-43384, NCCP-43385
	E154K	kappa (1.617.1)		NCCP-43389
	ΔE156/F157		delta (1.617.2)	NCCP-43390
	R158G		delta (1.617.2)	NCCP-43390
	R190S		gamma (P.1)	Twistbio-79683
	D215G		beta (B.1.351)	Twistbio-678597, NCCP-43382
417S	D253G	iota (B.1.526)		NCCP-43387
	K417T		gamma (P.1)	Twistbio-79683
	K417N		beta (B.1.351)	Twistbio-678597, NCCP-43382
	L452R	epsilon (B.1.427), epsilon (B.1.429), kappa (1.617.1)	delta (1.617.2)	NCCP-43384, NCCP-43385, NCCP-43389, NCCP-43390
484S	T478K		delta (1.617.2)	NCCP-43390
	E484K	eta (B.1.525), iota (B.1.526)	beta (B.1.351), gamma (P.1)	Twistbio-678597, NCCP-43382, NCCP-43386, NCCP-43387, Twistbio-79683
	E484Q	kappa (1.617.1)		NCCP-43389
	N501Y		alpha (B.1.1.7), beta (B.1.351), gamma (P.1)	Twistbio-601443, Twistbio-678597, Twistbio-710528, Twistbio-79683, NCCP-43381, NCCP-43382
570S	A570D		alpha (B.1.1.7)	Twistbio-710528, Twistbio-601443, NCCP-43381
	D614G	eta (B.1.525), iota (B.1.526), epsilon (B.1.427), epsilon (B.1.429), kappa (1.617.1)	alpha (B.1.1.7), beta (B.1.351), gamma (P.1), delta (1.617.2)	Twistbio-601443, Twistbio-710528, Twistbio-678597, Twistbio-79683, NCCP-43381, NCCP-43382, NCCP-43384, NCCP-43385, NCCP-43386, NCCP-43387, NCCP-43389, NCCP-43390
	H655Y	iota (B.1.526)	gamma (P.1)	Twistbio-79683, NCCP-43387
	Q677H	eta (B.1.525)		NCCP-43386
	P681H		alpha (B.1.1.7)	Twistbio-710528, Twistbio-601443, NCCP-43381
	P681R	kappa (1.617.1)	delta (1.617.2)	NCCP-43389, NCCP-43390
	A701V	iota (B.1.526)	beta (B.1.351)	Twistbio-678597, NCCP-43382, NCCP-43387
	T716I		alpha (B.1.1.7)	Twistbio-710528, Twistbio-601443, NCCP-43381
