# Supplementary material for: Development of an efficient Sanger sequencing-based assay for detecting SARS-CoV-2 spike mutations
Source: PLoS One. 2021 Dec 14;16(12):e0260850. doi: 10.1371/journal.pone.0260850 (PMC8670694; doi:10.1371/journal.pone.0260850)
Supplement: S2 Table — Abbreviations: SARS-CoV-2, Severe acute respiratory syndrome-related coronavirus 2; ATCC, American Type Culture Collection; KBPV, Korea Bank for Pathogen Viruses; NIBSC, National Institute for Biological Standards and Control; KCTC, Korean Collection for Type Cultures. (PDF) [file pone.0260850.s003.pdf]

Group	Strain	Source	PCR result
Virus	Dengue virus type 2	ATCC VR-1584	Negative
	Dengue virus type 4	ATCC VR-1490	Negative
	Herpes virus type 2	ATCC-VR-734	Negative
	Echovirus 30	KBPV-VR-25	Negative
	Human Adenovirus type 40	ATCC VR-931	Negative
	Human Parvovirus-B19	NIBSC-12/208	Negative
	BK virus	ATCC VR-837	Negative
	Coxsackie virus B3	ATCC VR-30	Negative
	Coxsackie virus B5	ATCC VR-185	Negative
Bacteria	*Enterococcus faecium*	KCTC-13225	Negative
	Enterococcus faecalis	KCTC-5290	Negative
	Staphylococcus Saprophyticus	KCTC-3345	Negative
	Streptococcus agalactiae	ATCC-12928	Negative
	Klebsiella quasipneumoniae sub	ATCC-700603	Negative
	Lactobasillus jensenii	ATCC-25258	Negative
	Lactobasillus gasseri	ATCC-33323	Negative
	Lactobasillus crispatus	ATCC-33820	Negative
	Proteus mirabilis	ATCC-29906	Negative
	Haemophillus ducreyi	ATCC-33940	Negative
