# Supplementary material for: Development of an efficient Sanger sequencing-based assay for detecting SARS-CoV-2 spike mutations
Source: PLoS One. 2021 Dec 14;16(12):e0260850. doi: 10.1371/journal.pone.0260850 (PMC8670694; doi:10.1371/journal.pone.0260850)
Supplement: Attachment — Submitted filename: Response to Reviewers.docx [file pone.0260850.s005.docx]

**Responses to Academic Editor Comments**

Thank you for submitting your manuscript to PLOS ONE. After careful consideration, we feel that it has merit but does not fully meet PLOS ONE’s publication criteria as it currently stands. Therefore, we invite you to submit a revised version of the manuscript that addresses the points raised during the review process.

I have received the reviews of your manuscript. While your paper addresses an interesting question, the reviewers stated several concerns about your study and did not recommend publication in its present form. The presentation as well as the readability of the manuscript need to be improved, since the manuscript as a whole is quite convoluted. The title of the manuscript implied that the PCR plus Sanger Sequencing provided the necessary information. However, this is not clearly conveyed in the main text. The abstract only mentioned the 5-SSG primer assay development. From the main text, the 5-SSG primer assay system does not include sequence, how do the authors determine the mutation detected, PCR product size? In addition, the quality of the language needs to be improved, there are quite a few awkward sentences and typos throughout the manuscript. Please have a fluent, preferably native, English-language speaker thoroughly copyedit your manuscript for language usage, spelling, and grammar.

**A:** Thank you for your valuable comment. To improve the quality of the manuscript's expression and readability, we requested a native-speaker proofreading service, and the revised manuscript has been resubmitted here. Also, as per your suggestion, the title has been changed to “Development of an efficient Sanger sequencing-based assay for detecting SARS-CoV-2 spike mutations", to more accurately include the essential information provided by PCR and Sanger sequencing. All additional corrections made in the manuscript (Line 4-5, 37, and 106-118). Electrophoresis was performed to determine the size of the PCR product, and the results are summarized in Tables 2 and S2. For reference, photos of the electrophoresis have been attached below.

**Specific comments:**

1. **Need to declare that Jeong-Eui Lee is the Seegene employee**

**A:** We have carefully reconsidered the issue of Jeong-Eui Lee as a co-author. Since his direct contribution to the paper is not entirely clear, we have decided to remove his authorship. We apologize for any confusion we may have caused by not initially making a more careful decision.

2. **Line 88 – 91, please rephrase for clarity. What really did the authors develop? PCR assay in conjunction with Sanger sequencing or just PCR assay?**

**A:** We have modified the manuscript’s passage to say, “PCR assay in conjunction with Sanger sequencing” (Line 89).

3. **Line 99, “redesigned or modified” from what?**

**A:** We have modified the sentence passage to state, ““modified from Global Initiative on Sharing Avian Influenza Data (GISAID) database, with a frequency cut-off > 1%, applied with degenerative or inosine to optimize melting temperature (Tm), avoid repetitive sequences, and include GC content > 65%, using Gene Runner (ver. 6.0) [20, 21].” (Line 98-101).

4. **e 117 – 120, awkward sentence, please rephrase for clarity. Also, are these viruses, bacteria and fungi selected causing respiratory infections? What is the selection criteria?**

**A:** We have chosen here to focus on all pathogen species for which standard stains could be obtained to compare analytical capabilities. Among them, the results were separated between those causing respiratory infection (Table 2), and other pathogens (Supplementary Table 2) for clarity.

5. **Line 119, suggest changing “2 other kinds of coronaviruses” to “Coronavirus OC43 & 229E”**

**A:** As per your suggestion, we have changed the sentence to “Coronavirus OC43 & 229E” (Line 131).

6. **Line 135 – 137, awkward sentences, please rephrase for clarity.**

**A:** As follow your suggestion, we have reworded the sentences for clarity (Line 144-145).

7. **Line 146 – 147, this sentence is confusing, please rephrase.**

**A:** The sentence has been rephrased (Line 156-158).

8. **Line 198 – 199, please rephrase for clarity.**

**A:** The passage has been rephrased (Line 209-210)

**Responses to Reviewer Comments**

**Reviewer #1’s comments and responses**

▶ Comment 1: Lim et al. have developed SARS-CoV-2 Spike protein primer sets to distinguish VOC and VOI as defined by WHO. The primers seem to work well for the VOC strains and some of the VOI strains. However, recently, lambda (June 14) and mu (August 30) have been newly designated. Since mu is designated after the submission date of August 6, authors can dismiss the VOI. The lambda (C.37) has 246_253delinsN deletion, which coincides with the reverse primer of 144S. Authors need to check whether the current primer design can detect the deletion in the lambda variant. Also, authors needs to share the raw data of the sequencing data prior to the publication.

**▶ Response to comment 1:** Thank you for your very important comments. We have checked whether the 5-SSG primer assay system can detect deletion in Mu and lambda variants. As you point out, the lambda (C.37) 246_253delinsN deletion is not amplified under the influence of 144S reverse primer, but it can be confirmed with the 417S primer set. In addition, there is no problem in confirming Mu (B. 1.621) mutations.

We have attached the figure below to support this information.

Since your comments are very important, so we will monitor the occurrence of mutations and continue to improve our primer sets. As your request, we have included a new supplementary figure of raw data for the sequencing results of the key mutants, and have enclosed a data file to share the NGS raw data in FASTA format.

**Reviewer #2’s comments and responses**

This study describes development and testing of five primer sets for detecting SARS-CoV-2 spike variants using Sanger sequencing. The manuscript is well written, and was a pleasure to read. The study appears robust, apt, and timely, especially considering its potential for application in clinical settings.

**I only have a few minor comments:**

**▶ Comment 1:** A few typos:

- line 38, 'detect nine', instead of 'detect of nine'

- line 43, 'ability of the 5-SSG primer', instead of 'ability of 5-SSG primer'. Same for lines 250, 273, 284

- line 81, 'methods', instead of 'method' be appropriate.

**▶ Response to comment 1:** All errors in manuscript have been corrected (Line 35, 41-42, 77, 263, 286, and 297)

**▶ Comment 2:** In the first paragraph of the results section (lines 204-214) and throughout the manuscript, the authors refer to 'mutants' as 'variants'. The term 'variant' in SARS-CoV-2 literature is probably more appropriately used for a constellation of mutations that make up a genetically (and usually epidemiologically) distint virus, rather than single mutations, and I fear that the authors use of the word here may not be appropriate.

**▶ Response to comment 2:** We agree with your valuable comment. The inappropriate words in manuscript have been corrected (Line 5, 6, 32, 42, 43, 74, 77, 101, 118, 119, 187, 216, 218, 219, 220, 221, 222, 223, 225, 273, 275, 276, 289, 290, 302, 311, 316, 324, 329, 341, and 345).

**▶ Comment 3:** Under acknowledgement, the name of the person being 'thanked' is missing.

**▶ Response to comment 3:** We have been added the name of Editage in acknowledgement (Line 359).
