# Supplementary material for: Development of an efficient Sanger sequencing-based assay for detecting SARS-CoV-2 spike mutations
Source: PLoS One. 2021 Dec 14;16(12):e0260850. doi: 10.1371/journal.pone.0260850 (PMC8670694; doi:10.1371/journal.pone.0260850)
Supplement: Attachment — Submitted filename: Response to reviewer.docx [file pone.0260850.s006.docx]

**Specific comments**

1. Line 135 - 136, "Anonymized residual of 17 nasopharyngeal swab specimens SARS-CoV-2 positive..." suggest changing to "Anonymized residual of 17 SARS-CoV-2 positive nasopharyngeal swab specimens..."

**A:** Thank you for your valuable comment. We have modified this text in the manuscript as “anonymized residual of 17 SARS-CoV-2 positive nasopharyngeal swab specimens of patients diagnosed with SARS-CoV-2 positive between February and June 2021 were obtained and used for this study” (lines 145-148).

2. Line 146 - 147, "The comparative LOD assessment was performed 25 times until the concentration of the targeted band was detected as positive." Not sure what the authors want to convey, please rephrase for clarity.

**A:** We have modified the sentence as follows for clarity: “For the performance analysis of 5-SSG primers, 25 replicates were performed. The comparative LOD was determined as the minimum detectable concentration” (lines 157-159).

3. Line 150 & 152, change PCR to RT-PCR.

**A:** As per your suggestion, we have changed the subheading and sentence to RT-PCR (lines 162, 164).

**Responses to Reviewer Comments**

**Reviewer #1’s comments and responses**

**▶ Comment:** **Authors have responded to my review comments on 246_253delinsN on lambda variant. However, since this is an important point, authors should address that their assay works for the lambda and others in discussion.**

**▶ Response to comment:** Thank you for your valuable comment. As suggested, we mentioned the lambda variant issue in this study, as the 246_253delinsN mutation, in the discussion section of the manuscript (lines 337-341).
